# Amyloid Goiter, Papillary Thyroid Carcinoma, and Diffuse Thyroid Lipomatosis: A Case Report of a Rare Association

**DOI:** 10.7759/cureus.71245

**Published:** 2024-10-11

**Authors:** Guilherme Vaz de Assunção, Liliana Fonseca, José R Brandão, André Couto de Carvalho, Cláudia Freitas

**Affiliations:** 1 Division of Endocrinology, Diabetes and Metabolism, Centro Hospitalar Universitário de Santo António, Unidade Local de Saúde de Santo António, Porto, PRT; 2 Department of Pathology, Genetics and Pathology Clinic, Centro Hospitalar Universitário de Santo António, Unidade Local de Saúde de Santo António, Porto, PRT

**Keywords:** amyloid goiter, amyloidosis, case report, diffuse thyroid lipomatosis, enlarged thyroid, papillary thyroid carcinoma, ptc

## Abstract

Amyloid goiter is an extremely rare condition. It results from amyloid infiltration of the thyroid, typically associated with AL (primary) or AA (secondary) amyloidosis, and has occasionally been linked to diffuse fatty infiltration or papillary thyroid carcinoma. The combination of these three conditions is exceptionally rare.

We present a case of a 54-year-old male with chronic refractory gout on immunosuppressive therapy who developed a multinodular goiter. A thyroid ultrasound revealed a 45-mm solid hypoechoic nodule classified as European Thyroid Imaging Reporting and Data System 4 (EU-TIRADS 4). Thyroid function was normal. After two non-diagnostic fine needle aspirations, clinical surveillance was chosen. Four years later, the patient presented with thyroid enlargement accompanied by compressive symptoms, which were confirmed as tracheal compression on imaging. A total thyroidectomy revealed amyloid deposition, diffuse lipomatosis, and multifocal papillary microcarcinomas (dominant 0.2 cm). Postoperatively, the investigation for systemic amyloidosis was negative, and the patient demonstrated an excellent clinical and biochemical response, with no recurrence of amyloid deposits.

To the best of our knowledge, this is the fourth reported case in the literature of amyloid goiter associated with papillary carcinoma and diffuse thyroid lipomatosis. The case underscores the importance of considering amyloid goiter in patients with significant thyroid enlargement, especially those with chronic inflammatory conditions. Histopathological evaluation is key, and further research is needed to explore potential links between chronic inflammation, amyloid deposition, and thyroid cancer development.

## Introduction

Amyloidosis is a condition characterized by the extracellular deposition of insoluble protein fibrils, known as amyloid, in various organs and tissues, potentially leading to severe organ dysfunction [1,2]. It can be systemic or localized, with systemic forms affecting multiple organ systems [2]. The most frequent types of amyloidosis include AL (due to light chain fragment, also known as primary amyloidosis), ATTR (amyloid transport protein transthyretin), and AA (due to serum amyloid A protein, also known as secondary amyloidosis) [2-4].

Amyloid goiter is an extremely rare manifestation of amyloidosis, characterized by the deposition of amyloid proteins within the thyroid gland [1,3,5]. This condition typically arises in the context of systemic amyloidosis but may also present as an isolated thyroid manifestation. Clinically, patients present with a rapidly enlarging, non-tender thyroid mass, which can cause compressive symptoms such as dyspnea, dysphagia, and hoarseness [1,6]. The rarity and nonspecific symptoms often complicate the diagnosis of amyloid goiter, requiring histopathological examination for confirmation [5,6].

In contrast to the rarity of amyloid goiter, papillary thyroid carcinoma (PTC) represents the most prevalent type of thyroid cancer [7]. It is characterized by follicular cell differentiation and distinctive nuclear features. Frequently, incidental findings of PTC occur, especially with the ones with 1 cm or less (called microcarcinomas). Although some subtypes are associated with a worse outcome, generally, PTC carries an excellent prognosis with a low risk of progression or metastasis. Management of PTC is individualized, ranging from active surveillance in low-risk cases to surgical resections such as thyroidectomy or lobectomy, depending on the patient's risk factors and preferences [7,8].

Another rare condition affecting the thyroid gland is diffuse thyroid lipomatosis. This condition is marked by the abnormal accumulation of adipose tissue within the thyroid, resulting in glandular enlargement and frequently causing compressive symptoms [8,9]. The pathogenesis of diffuse thyroid lipomatosis remains poorly understood and is often incidentally detected during imaging or surgical procedures [10,11].

This case report describes the unusual coexistence of amyloid goiter, PTC, and diffuse thyroid lipomatosis. The concurrence of these conditions underscores the complexity of thyroid pathology and highlights the importance of comprehensive diagnostic evaluation in optimizing patient outcomes.

## Case presentation

We report the case of a 54-year-old male with a history of chronic refractory tophaceous gout, hypertension, chronic kidney disease (G3aA1 stage, Kidney Disease: Improving Global Outcomes classification), obesity class II with a body mass index of 36.0 Kg/m², obstructive sleep apnea syndrome, and hypertensive cardiomyopathy associated with New York Heart Association class II heart failure with preserved ejection fraction. His routine medication regimen included anakinra 100 mg once a day (QD), prednisolone 5 mg QD and febuxostat 80 mg QD for gout management, losartan 50 mg QD, and amlodipine 5 mg QD. No other significant pathologies were noted, including any known thyroid pathology.

**Figure 1:**
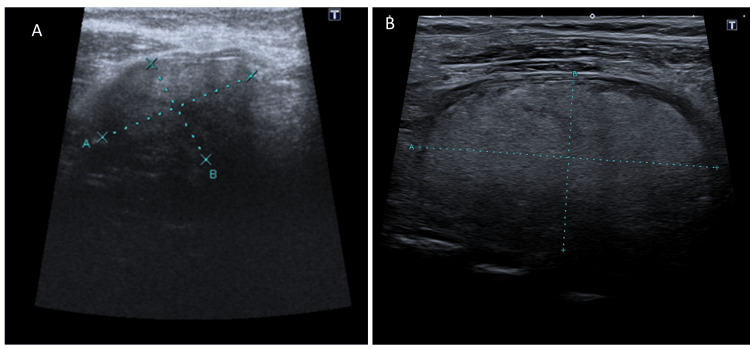
The thyroid ultrasound revealed a notable nodule (EU-TIRADS 4) measuring 45x28 mm in the right lobe (A), and its evolution after four years (B), measuring 59x33 mm. (anteroposterior × width) EU-TIRADS: European Thyroid Imaging Reporting and Data System

**Figure 2:**
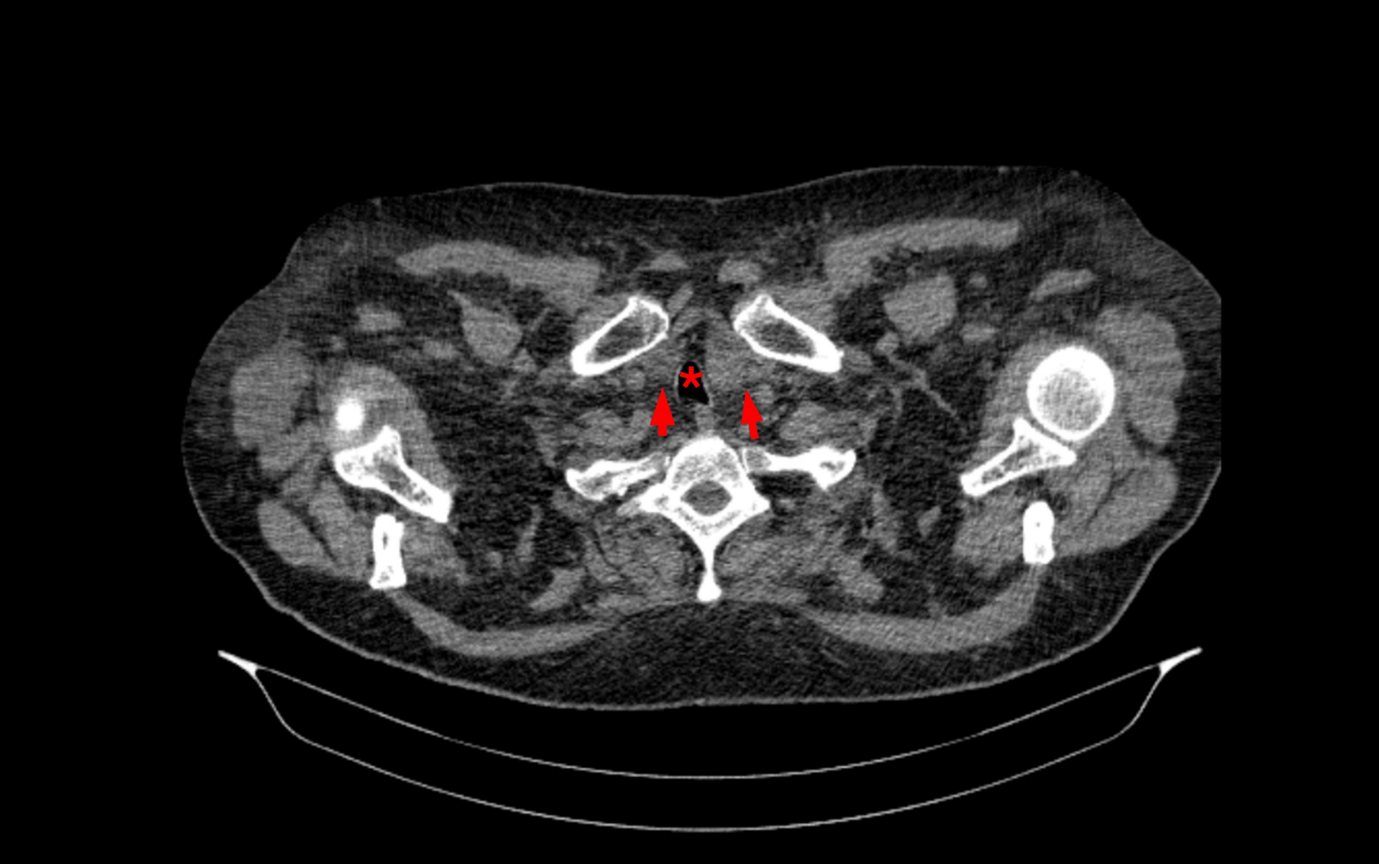
Cervical-thoracic CT scan reveals thyroid enlargement, denoting retrosternal goiter (red arrows) and tracheal compression (red "asterisk" - trachea).

In 2015, he was referred to our Endocrinology/Thyroid Outpatient Clinic due to an enlarged thyroid, notable on the ultrasound (US) for a solid hypoechoic nodule, measuring 45 mm of largest diameter, located at the junction of the right lobe and the isthmus, and classified as European Thyroid Imaging Reporting and Data System 4 (EU-TIRADS 4) (Figure 1). Additionally, there were nodules in the left lobe: one at the transition with the isthmus measuring 26 mm of largest diameter and another at the inferior pole measuring 37 mm of largest diameter, both classified as low-risk EU-TIRADS 2. Thyroid function revealed a thyroid-stimulating hormone (TSH) level of 1.50 µIU/mL (reference range 0.30 - 3.18), and anti-thyroglobulin and anti-thyroid peroxidase antibodies were both negative. A fine-needle aspiration cytology (FNAC) of the dominant right-isthmus was performed, yielding a nondiagnostic result (Bethesda I). Following two months, FNAC was repeated, resulting in the same category. Upon discussion with the patient and considering the lack of compressive symptoms, he was maintained under active surveillance. After four years, during a follow-up examination, neck compressive symptoms with the notion of further thyroid enlargement were reported. Thyroid US (Figure 1) revealed thyroid nodule growth, with the largest in the right lobe measuring 59x33 mm (anteroposterior × width) and the other two on the left with 39 and 31 mm. Updated thyroid tests revealed a normal function. Upon performing a computed tomography (CT) scan, an enlarged thyroid was observed, causing a mass effect on adjacent structures along with evident compression of the trachea (Figure 2).

**Figure 3:**
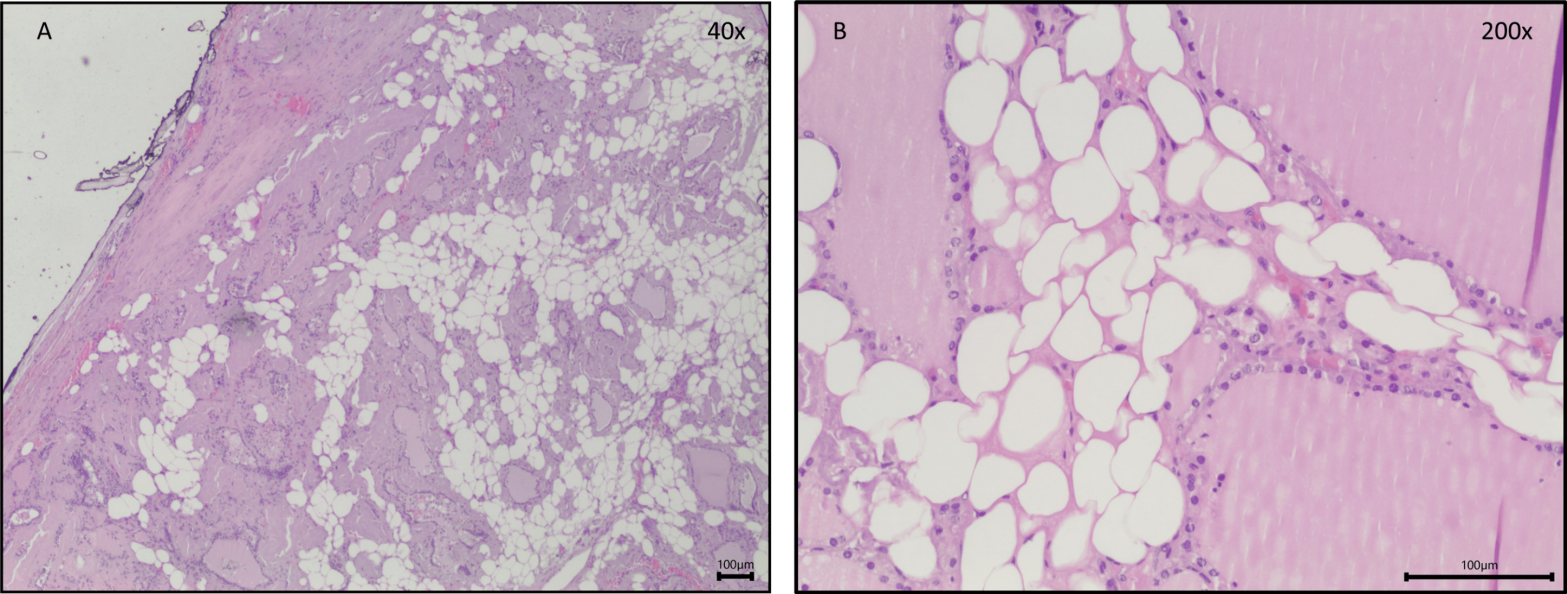
Histopathological features of the excised thyroid gland. Photomicrographs of parenchyma with areas of mature adipose tissue and acellular, amorphous, and eosinophilic material (A and B: Hematoxylin and Eosin). Objective magnification: A x40 and B x200.

**Figure 4:**
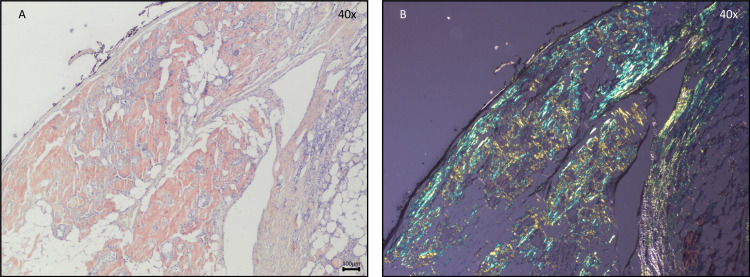
Histopathological features of the excised thyroid gland. Photomicrographs of parenchyma with areas of adipose tissue and acellular, amorphous, and eosinophilic material that is also congophilic by the Congo red (A: Congo red stain) and birefringence apple-green under polarized light (B: Congo red stain under polarized light). Objective magnification: x40.

**Figure 5:**
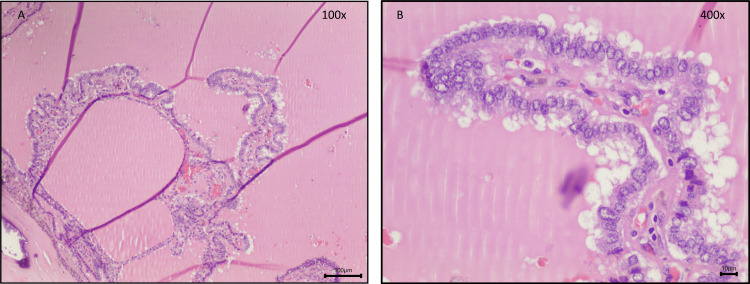
Histopathological features of the excised thyroid gland. Photomicrographs demonstrating one of the foci of papillary carcinoma of the classical subtype, with papillary areas associated with dystrophic calcification and papillary pattern with cells containing nuclear features of papillary carcinoma, namely, large size with crowding, chromatin margination (Orphan Annie-eye), and irregular contours and grooves (A, B). No intranuclear pseudo-inclusions are seen in this image (B) (A and B: Hematoxylin and Eosin). Objective magnification: A x100 and B ×400.

Given this progressive thyroid enlargement and the onset of compressive symptoms, the patient underwent a total thyroidectomy. The excised thyroid gland weighed 125 grams and measured 9.5×6.0×4.5 cm for the right lobe, 8.0×5.0×3.0 cm for the left lobe, and 3.0×2.0×0.5 cm for the isthmus. Histopathological examination revealed parenchyma with diffuse areas of adipose tissue and acellular, amorphous, and eosinophilic material (Figure 3) that were also congophilic by Congo red and showed birefringence apple-green under polarized light, revealing amyloid deposition (Figure 4). In addition, multiple foci of bilateral papillary thyroid microcarcinomas (pT1a multifocal in accordance with the 8^th^ edition of the TNM Staging System for Differentiated Thyroid Cancer) were identified, with the largest measuring 2 mm in diameter, with some papillary areas and typical papillary nuclear features associated with dystrophic calcification and no evidence of lymphovascular invasion or microscopic extrathyroidal extension (Figure 5). These findings indicated an incidental papillary thyroid microcarcinoma occurring in the context of significant amyloid infiltration and diffuse lipomatosis of the thyroid gland.

Assuming a very low risk of recurrence, levothyroxine therapy was started, with the goal of maintaining TSH levels in the 0.5-2.0 µIU/mL range. On follow-up, the patient demonstrated an excellent biochemical and structural response with no evidence of residual malignant thyroid disease. Regarding the amyloid goiter, he underwent extensive investigation, but other potential sources of amyloidosis, besides gout, returned negative. No other organs have shown manifestations, and the patient has not demonstrated any evidence of recurrent amyloid disease.

## Discussion

The coexistence of amyloid goiter, PTC, and diffuse thyroid lipomatosis in a single patient is extraordinarily rare. Individually, each of these conditions is uncommon. Amyloid goiter, characterized by the deposition of amyloid within the thyroid gland, is an infrequent manifestation of systemic or localized amyloidosis, typically associated with chronic inflammatory conditions, such as chronic kidney disease or autoimmune disorders [2,4]. PTC, although a common type of thyroid cancer, has an exceptional occurrence alongside amyloid deposition and/or lipomatosis [8,11]. Diffuse thyroid lipomatosis, marked by the infiltration of mature adipose tissue within the thyroid gland without encapsulation, is a rare entity, with few cases documented in the literature [9,10]. The simultaneous presence of these three conditions in one patient not only underscores the rarity of this case but also presents significant diagnostic and therapeutic challenges, emphasizing the need for a comprehensive approach.

A review of the literature reveals very few cases reporting the coexistence of amyloid goiter, PTC, and diffuse thyroid lipomatosis. Most reported cases involve the coexistence of one or two of these conditions, but rarely all three in combination. To our knowledge, only three other case reports describe a similar combination of these pathologies (Table 1).

**Table 1:** Patient characteristics and diagnostic work-up findings for cases of amyloid deposition, papillary thyroid carcinoma, and diffuse thyroid lipomatosis as reported in the literature. Dimensions: anteroposterior × width × length ISM: ishtmus; LL: left lobe; LT: levothyroxine; MRI: magnetic resonance imaging; RAI: radioactive iodine; RL: right lobe; TC: computed tomography; US: ultrasound; y/o: years-old

Case reports	Sex, Age	Medical history	History of thyroid dysfunction	Imaging tests	Fine-needle aspiration results	Thyroidectomy specimen dimensions	Histologic features
Coli et al. (2000) [12]	Female, 74 y/o	Renal failure of unknown etiology.	Unavailable history of thyroid disease. Thyroid goiter.	Unavailable	Unavailable	Unavailable	Extensive adipose thyroidal metaplasia, amyloid A immunohistochemistry, papillary carcinoma.
Morado da Silva et al. (2022) [11]	Female, 54 y/o	Neurogenic bladder, chronic kidney disease due to chronic pyelonephritis, bronchiectasis with multiple respiratory infections.	No past history of thyroid disease. Presented with goiter with compressive symptoms for 4 months. Normal thyroid function.	US: Enlarged thyroid with one nodule solid, isoechoic, taller than wide, no calcifications, 28×19×29 mm. TC: Enlarged thyroid, reduction of tracheal diameter, areas suggestive of diffuse lipomatosis.	Bethesda IV category	Weight of 81 g. LL: 3.2×6.7×2.8 cm; ISM: 2.6×4.5×2.0 cm; RL: 4.5×7.5×3.4 cm.	Mature adipose tissue, amorphous eosinophilic material orangeophilic with Congo red and apple-green birefringence under polarized light confirming amyloid deposition, papillary macrocarcinoma of follicular variant; later confirmed to be systemic AA amyloidosis.
George and Shah (2024) [8]	Female, 51 y/o	Chronic kidney disease, amyloid nephropathy.	Toxic multinodular goiter known for 4 years and treated with RAI, under LT for 2 years afterward. Incidental finding of an enlarged thyroid.	MRI: Homogenously low-density mass of the neck with retrosternal and hypopharyngeal extension with bilateral displacement of the carotid sheath.	Unavailable	LL: 8.5x5.2x4.7 cm; ISM: 2.2x1.2x1.0 cm; RL: 9.4x6.6x5.7 cm; diffusely yellowish with focal grey-white and reddish-brown congested areas.	Lobules of adipocytes of varying sizes with atrophic thyroid follicles. Patchy foci of amorphous pink material, polarizes with apple-green birefringence on Congo red stain confirming amyloid deposition; papillary carcinoma with papillary cores, nuclear grooving, nuclear overlapping, intranuclear cytoplasmic inclusions, and psammomatous calcifications.
Current Case (2024)	Male, 54 y/o	Chronic refractory tophaceous gout, hypertension, chronic kidney disease, obesity class II, obstructive sleep apnea syndrome, hypertensive cardiomyopathy, heart failure.	Multinodular goiter. Presented with compressive symptoms after 4 years. Normal thyroid function.	US: Enlarged thyroid, solid hypoechoic RL nodule 45 mm of largest diameter; LL: One nodule 26 mm of largest diameter and another 37 mm of largest diameter. CT scan denoting retrosternal goiter and tracheal compression.	Bethesda I category	Weight of 125 grams; RL: 9.5×6.0×4.5 cm; ISM: 3.0×2.0×0.5 cm; LL: 8.0×5.0×3.0 cm.	Parenchyma with areas of adipose tissue and acellular, amorphous, and eosinophilic material also congophilic by Congo red and showed birefringence apple-green under polarized light, revealing amyloid deposition; multiple foci of bilateral papillary thyroid (largest 2 mm).

In a case reported by George et al., the authors described an amyloid goiter with diffuse thyroid lipomatosis and an incidental finding of PTC. Their patient presented with a massive enlarged thyroid and a history of chronic inflammatory disease, similar to our case and others reported in the literature. Regrettably, the reported medical history is unclear about the type of amyloidosis [8]. Most of the cases of amyloid goiter are associated with AA amyloidosis; therefore, a previous diagnosis in an individual with confirmed amyloidosis could help raise the suspicion of amyloid goiter in the presence of enlarged goiter and compressive signs [13]. This case also underscores the diagnostic challenge posed by the incidental detection of PTC amidst significant thyroid pathology, with carcinomas only identified incidentally after surgery [8,11,14].

Another case described by Silva et al. involved a 54-year-old woman with papillary thyroid carcinoma, secondary amyloid goiter, and diffuse thyroid lipomatosis [11]. In contrast to the present case, this report by Silva et al. had a rapid enlargement of the goiter with compressive symptoms within a span of a few months with no apparent known history of thyroid disease [11]. A rapid onset of compressive symptoms is commonly described, although there are also cases of slower growth rates [13]. For our patient, it took approximately four years from the recognition of the multinodular goiter to the onset of compressive symptoms. Unfortunately, due to the absence of studies detailing median times for slow or rapid progression in the context of amyloid goiters, we are unable to categorize our patient as either. A further difference from the Silva et al. case is the diagnosis of systemic AA amyloidosis, which is yet to be confirmed in our patient. Nonetheless, both reports highlight the importance of surgical management, given the presence of compressive symptoms and the need for a definitive diagnosis [11].

The co-occurrence of these three distinct thyroid conditions could suggest the possibility of underlying shared pathophysiological mechanisms. Chronic inflammation has been implicated in the pathogenesis of both amyloidosis and thyroid malignancies [4,15]. In the present case, the patient’s chronic gout likely contributed to persistent low-grade inflammation, and even though we do not have a clear immunohistochemical distinction between AL or AA amyloid, this may have facilitated the development of localized amyloid deposition within the thyroid gland.

The diagnostic process in our case was particularly challenging. FNAC of the single suspicious EU-TIRADS 4 nodule was nondiagnostic (Bethesda I) in two separate moments. This is a widely used diagnostic tool for evaluating thyroid nodules, but its utility may be limited in cases of amyloid goiter due to the dense, amorphous nature of amyloid deposits, which can result in inadequate sampling and nondiagnostic results [6,16].

Ultimately, a total thyroidectomy was performed, which not only helped the compressive symptoms but also allowed a thorough histopathological assessment, leading to its definitive diagnosis. At the last follow-up, the patient demonstrated an excellent clinical and biochemical response with no evidence of malignancy or amyloid recurrence, confirming the appropriateness of surgical intervention. This outcome highlights the importance of surgical management in complex cases where multiple thyroid pathologies coexist, particularly when compressive symptoms or the potential for malignancy are present [3,8,11].

## Conclusions

To the best of our knowledge, this case presents the fourth report of a rare thyroid coexistence of amyloid goiter, papillary thyroid carcinoma, and diffuse thyroid lipomatosis. Chronic refractory gout likely contributed to the amyloid deposition within the thyroid, while the limitations of fine-needle aspiration cytology in detecting amyloid further delayed its diagnosis. Total thyroidectomy was crucial for enabling a definitive conclusion. The patient’s excellent postoperative response, with no recurrence of amyloid or malignancy, highlights the importance of surgical intervention in the thyroid, particularly when neck compressive symptoms or malignancy are suspected. Additional studies are warranted to investigate potential common pathophysiological pathways, with a particular focus on the impact of chronic inflammation and amyloid deposition in the progression of thyroid diseases.
